# Impact of a Heterozygous C1R^R301P/WT^ Mutation on Collagen Metabolism and Inflammatory Response in Human Gingival Fibroblasts

**DOI:** 10.3390/cells14070479

**Published:** 2025-03-22

**Authors:** Chengjuan Qu, Cecilia Koskinen Holm

**Affiliations:** Department of Odontology, Umeå University, 90185 Umeå, Sweden; cecilia.koskinen@umu.se

**Keywords:** periodontal Ehlers–Danlos syndrome, heterozygous C1R^R301P/WT^-mutated human TERT-immortalized gingival fibroblasts (mhGFBs), complement component 1r/1s (C1r/C1s), collagen metabolism

## Abstract

Periodontal Ehlers–Danlos syndrome arising from heterozygous pathogenic mutation in *C1R* and/or *C1S* genes is an autosomal-dominant disorder characterized by early-onset periodontitis. Due to the difficulties in obtaining and culturing the patient-derived gingival fibroblasts, we established a model system by introducing a heterozygous C1R^R301P/WT^ mutation into human TERT-immortalized gingival fibroblasts (hGFBs) to investigate its specific effects on collagen metabolism and inflammatory responses. A heterozygous C1R^R301P/WT^ mutation was introduced into hGFBs using engineered prime editing. The functional consequences of this mutation were assessed at cellular, molecular, and enzymatic levels using a variety of techniques, including cell growth analysis, collagen deposition quantification, immunocytochemistry, enzyme-linked immunosorbent assay, and quantitative real-time reverse transcription polymerase chain reaction. The C1R^R301P/WT^-mutated hGFBs (mhGFBs) exhibited normal morphology and growth rate compared to wild-type hGFBs. However, mhGFBs displayed upregulated procollagen α1(V), MMP-1, and IL-6 mRNA expression while simultaneously downregulating collagen deposition and C1r protein levels. A modest accumulation of unfolded collagens was observed in mhGFBs. The mhGFBs exhibited a heightened inflammatory response, with a more pronounced increase in MMP-1 and IL-6 mRNA expression compared to TNF-α/IL-1β-stimulated hGFBs. Unlike cytokine-stimulated hGFBs, cytokine-stimulated mhGFB did not increase *C1R*, *C1S*, procollagen α1(III), and procollagen α1(V) mRNA expression. Our results suggest that the C1R^R301P/WT^ mutation specifically disrupts collagen metabolism and inflammatory pathways in hGFBs, highlighting the mutation’s role in these processes. While other cellular functions appear largely unaffected, these findings underscore the potential of targeting collagen metabolism and inflammation for therapeutic interventions in pEDS.

## 1. Introduction

Periodontal Ehlers–Danlos syndrome (pEDS) is a type of the genetic disorder EDS, which affects connective tissue [1,2]. The main feature of pEDS is severe and early-onset periodontitis, typically beginning around puberty [2,3,4,5,6]. Patients with pEDS also exhibit fragile, receding gingiva, easy bruising, and pre-tibial hyperpigmentation [2,7,8,9].

Unlike other forms of EDS, which typically involve collagen mutations or enzymes involved in their biosynthesis, pEDS arises from heterozygous pathogenic mutations in the *C1R* and/or *C1S* genes, located on chromosome 12 in region p13 [7,10,11]. These genes encode proteins C1r and C1s, subunits of the complement C1 complex, which is involved in the initial step of the classical complement pathway, a crucial part of the immune system’s defense against pathogens [7,12,13,14].

Previously, the C1R and/or C1S mutations in pEDS were thought to be gain-of-function mutations, leading to hyperactive proteins and excessive complement pathway activation [13,15,16]. However, a recent study by Amberger and his colleagues using primary skin fibroblasts from pEDS patients suggests a more nuanced picture. This study revealed that pEDS involves not only complement overactivation but also impaired degradation of matrix proteins by C1s [16], highlighting a dual mechanism.

Investigating the detailed molecular mechanisms of these mutations in gingival fibroblasts from individuals with pEDS would provide valuable insights for patient treatment. However, obtaining and culturing these cells remains challenging due to their relatively short lifespan, which restricts their use in research. Fibroblast immortalization extends cellular lifespan. Studies suggest that TERT-immortalized fibroblasts retain key primary cell characteristics, such as collagen metabolism and gene expression profiles [17,18,19,20]. This makes them a valuable, consistent, reproducible, and practical model for studying the genetic and molecular mechanisms underlying pEDS, particularly for investigating specific mutations, as they allow for precise genetic manipulation. However, immortalization may alter certain cellular behaviors, including collagen metabolism and C1r/C1s expression. Therefore, their ability to fully replicate pEDS-specific gingival pathology remains uncertain and requires further validation.

Previous research has revealed a higher prevalence of C1R mutation (12 families) compared to C1S mutation (two families) in pEDS patients. Among C1R mutations, the c.902G > C (p.Arg301Pro) has been frequently observed in pEDS individuals [7]. Given the frequency and potential significance of this mutation, and to address the limitations of existing research, we specifically introduced a heterozygous C1R^R301P/WT^ mutation into human TERT-immortalized gingival fibroblasts (hGFBs) using prime editing.

Using the mutated hGFBs (mhGFBs) model, we employed immunocytochemical staining (ICC), enzyme-like immunosorbent assay (ELISA), real-time quantitative polymerase chain reaction (RT-qPCR), and collagen deposition assays to investigate collagen metabolism and C1r/C1s expression. Our central hypothesis is that the heterozygous C1R^R301P/WT^ mutation in hGFBs alters collagen metabolism and dysregulates C1r/C1s expression under inflammatory conditions, contributing to the pathophysiology of pEDS. This study aims to (1) determine how the C1R^R301P/WT^ mutation affects C1r/C1s expression and function and (2) elucidate its impact on collagen degradation and inflammatory responses in gingival fibroblasts. These findings may provide insights for improved diagnosis and potential therapeutic targets for pEDS.

## 2. Materials and Methods

### 2.1. Cell Source and Culture

The hGFBs (ATCC, Manassas, VA, USA) were cultivated with fibroblast culture medium (FB medium: Dulbecco’s Modified Eagle Medium (DMEM, Gibco, Waltham, MA, USA) supplemented with 10% fetal bovine serum (FBS, Gibco) and 1% penicillin and streptomycin (Gibco)) in 2-dimensional monolayer (2D) culture in a humidified incubator at 37 °C with 5% CO_2_. At 80–90% confluency, the cells were either passaged in new flasks (Thermo Fisher Scientific, Waltham, MA, USA) for expansion or seeded in specific plates for various experiments as follows: 24-well plates (Thermo Fisher Scientific) for cell proliferation, collagen deposition assay, or immunocytochemical staining (ICC) and 6 cm dishes (Thermo Fisher Scientific) for cell growth, cell size, and cell population doubling time (PDT) detection over 24, 48, 72, 96, and 120 h. The cells were also seeded in 24- or 12-well plates and cultured with FB medium (used as control) or treated with 50 ng/mL of recombinant human tumor necrosis factor alpha (TNF-α) (R&D system, Minneapolis, MA, USA) or 100 pg/mL of recombinant human interleukin-1 beta (IL-1β) (R&D system) for collagen deposition or protein/gene expression assay.

### 2.2. Introducing a Heterozygous C1R^R301P/WT^ Mutation into hGFBs and Their Cultivation

To investigate the effects of a C1R-mutation, we engineered a heterozygous C1R-R301P mutation into hGFBs using prime editing. Briefly, we employed the enhanced prime editing system PE4 and a custom-designed prime editing guide RNA (pegRNA) [21,22] targeting the *C1R* gene. The pegRNA also incorporated a HindIII restriction site to facilitate genotyping.

A co-selection strategy with ouabain (Sigma-Aldrich, St. Louis, MO, USA) was used to enrich edited cells. The hGFBs were co-electroporated with equimolar amounts of pegRNA plasmid (containing an ATP1A1-Q118R mutation), pCMV-PEmax plasmid, and pEF1a-hMLH1dn plasmid (Addgene, Watertown, NY, USA) using the Neon system (Thermo Fisher Scientific). Ouabain resistance, conferred by the ATP1A1-Q118R mutation, served as a selectable marker for the edited cells [23,24].

Following selection, Sanger sequencing of cloned C1R polymerase chain reaction (PCR) products and droplet digital PCR (ddPCR, QX200 System, Hercules, CA, USA) were performed to confirm the presence and frequency of the C1R^R301P/WT^ mutation in individual clones. The resulting cells, mhGFBs, were heterozygous for the C1R^R301P/WT^ mutation.

The mhGFBs were cultivated identically to hGFBs under standard 2D culture using FB medium. Once reaching 80–90% confluency, cells were either passaged for expansion or used in downstream experiments (cell growth, proliferation, PDT, ICC, collagen deposition, and protein/gene expression assays), as previously described for hGFBs.

### 2.3. Cell Proliferation Assay

Cell proliferation of mhGFBs and hGFBs was detected using 3-(4,5-dimethylthiazol-2-yl)-2,5-diphenyltetrazolium bromide (MTT, Sigma-Aldrich) colorimetric assay. Briefly, hGFBs or mhGFBs from 3 clones were cultured for 48 h and then incubated with 0.5 mg/mL MTT reagent for three hours at 37 °C. After washing, formazan crystals were dissolved in a 1:1 (*v*/*v*) solution of dimethyl sulfoxide (Sigma-Aldrich) and ethanol. Absorbances of the dissolved formazan at 595 nm were measured using a 96-well plate reader (Sarstedt, Newton, NC, USA). Each sample was analyzed in triplicate. The experiments were repeated with at least three independent cell passages.

### 2.4. Cell Growth, Size, and Morphology Assessment

The mhGFBs or hGFBs were cultured for 24, 48, 72, 96, and 120 h. After trypsinization with trypsin/EDTA (Gibco), the cell number and size were determined using a Countess Automated Cell Counter (Invitrogen, Waltham, MA, USA). The PDT was calculated based on 72-h cell counts. The experiment was repeated at least three times with independent cell passages. Cell morphology was monitored throughout the culture period.

### 2.5. Immunocytochemical Staining and Quantification

The mhGFBs or hGFBs were grown on coverslips (Paul Marienfeld, Lauda-Königshofen, Germany) in 24-well plates. At 50–60% confluence, cells were fixed with 4% paraformaldehyde and either permeabilized with 0.1% Triton X-100 (Merck, Rahway, NJ, USA) for filamentous actin staining with Alexa FluorTM 488 Phalloidin (1:200, Thermo Fisher Scientific), vimentin staining with anti-vimentin antibody (EPR3776) (1:500, Abcam, Cambridge, UK), cluster of differentiation 90 (CD90) staining with mouse anti-THY-1, (1:200, Millipore, Burlington, MA, USA), and platelet-derived growth factor receptor alpha (PDGFRα) staining with human PDGFR alpha antibody (1:50, R&D systems), or left non-permeabilized for collagen I staining with an anti-collagen I antibody (1:500, Abcam) or unfolded collagens staining with 5 µM of fluorescence-collagen hybridizing peptide (F-CHP, 3Helix, Salt Lake City, UT, USA) overnight at 4 °C. After washes, the cells were incubated with Texas red goat anti-rabbit or Texas red goat anti-mouse secondary antibody or fluorescein anti-goat (1:200, Vector Laboratories, Newark, CA, USA) to visualize the stained vimentin, CD90, PDGFRα, and collagen I. Then, nuclei were counterstained with 4′6′-Diamidino-2-phenylindole dihydrochloride (DAPI, 1 µg/mL, Sigma-Aldrich). Finally, the stained cells were mounted on a glass slide with antifade mounting medium (Vector Laboratories) and imaged using a fluorescence microscope (Olympus BX41, Tokyo, Japan) or a Leica DM 6000 B microscope (Leica Microsystems, Wetzlar, Germany). Cultures incubated with PBS instead of anti-vimentin, anti-CD90 or anti-PDGFRα primary antibody were served as negative controls.

ImageJ software (version 1.53e) was used to quantify type I collagen and unfolded collagen fluorescence intensity in mhGFBs and hGFBs after subtracting DAPI fluorescence intensity. Ten different images per cell type were analyzed and averaged.

### 2.6. Collagen Deposition Assay

The mhGFBs or hGFBs were first cultured with 100 µM of L-ascorbic acid (Merck) to aid collagen synthesis. Then, they were treated with TNF-α, IL-1β, or control medium (FB medium) for 24 h. Subsequently, the collagen content was determined with in situ measurement of collagen synthesis with Sirius Red-based colorimetric microassay [25]. As previously described [26], the cells were fixed, permeabilized, and stained with 0.1% Sirius Red F3BA (Sigma-Aldrich) in a saturated picric acid (Sigma-Aldrich) solution. Unbonded dyes were removed with 10 mM of hydrochloride (VWR, Radnor, PA, USA) wash, and collagen-bound dye was solubilized with 0.1 M sodium hydroxide (AkzoNobel, Amsterdam, Netherlands) and measured at 540 nm using a 96-well plate reader. Each group was analyzed in triplicate, and the experiment was repeated with at least three independent cell passages.

### 2.7. C1r and C1s Protein Analysis by Enzyme-like Immunosorbent Assay (ELISA)

Secreted C1r and C1s protein levels were measured in conditioned media from 24-h cultures of mhGFBs and hGFBs. Media were frozen at −80 °C before analysis.

Sandwich ELISA kits (Human C1R (C1S)/Complement Component C1r (C1s) ELISA kit, Thermo Fisher Scientific) were used to quantify C1r and C1s protein according to the manufacturer’s instruction. Samples were analyzed in duplicate (100 µL/well) at 450 nm using a spectrophotometer (Multiskan GO, Thermo Fisher Scientific). Standard curves with recombinant human C1r or C1s proteins were used to calculate protein quantity (ng). Experiments were performed at least three times with independent cell passages.

### 2.8. Real-Time Quantitative Reverse Transcription Polymerase Chain Reaction (RT-qPCR)

Total ribonucleic acid (RNA) was extracted from mhGFBs and hGFBs cultured with FB medium (control), TNF-α, or IL-1β for 24 h. RNA quantity was measured and reverse transcribed to complementary DNA, as described previously [27]. The RT-qPCRs were performed, as previously described [27], with specific primers for target genes (*C1R* [28], *C1S* [28], vimentin [29], interleukin-6 (IL-6) [30], procollagen α1(I) [31], procollagen α1(III) [31], procollagen α1(V) [32], matrix metalloproteinase-1 (MMP-1) [33], tissue inhibitor of metalloproteinase-1 (TIMP-1) [33]), and a housekeeping gene, ribosomal protein lateral stalk subunit P0 (RPLP0) [34] (Table 1). The RT-qPCR cycling conditions were 95 °C for two minutes (initial denaturation), followed by 40 cycles of 95 °C for two seconds (denaturation) and 60 °C for 20 or 30 s (annealing).

Primer efficiency (90–110%) was determined by standard curves of each primer. Relative gene expression of all target genes was calculated using the Pfaffl method [35] with RPLP0 normalization. Specificity of PCR products was confirmed by post-PCR melting curve analysis and 2% (*w*/*v*) agarose gel electrophoresis separation. All experiments were repeated at least three times with independent RNA isolations.

### 2.9. Statistical Analysis

Statistical analyses were performed using SPSS software (IMB SPSS Statistics 29, Chicago, IL, USA). All data were analyzed by mean ± SD from at least three independent experiments. Data were tested for normality using the Shapiro–Wilk test for all data, thereafter, comparisons between different groups were performed with an independent-sample T-test for cell growth, size, PDT, and C1r/C1s protein. For the rest of the data, a non-parametric assay with Kruskal–Wallis H or Mann–Whitney U was used. *p*-values less than 0.05 were considered statistically significant.

## 3. Results

### 3.1. Generation of the Heterozygous C1R^R301P/WT^-Mutated hGFBs (mhGFBs)

We engineered mhGFBs using prime editing with co-selection. Briefly, we employed the PE4 prime editing system and pegRNAs [21] to target one allele of the *C1R* gene in hGFBs. A HindIII restriction site was incorporated to simplify genotyping.

**Figure 1:**
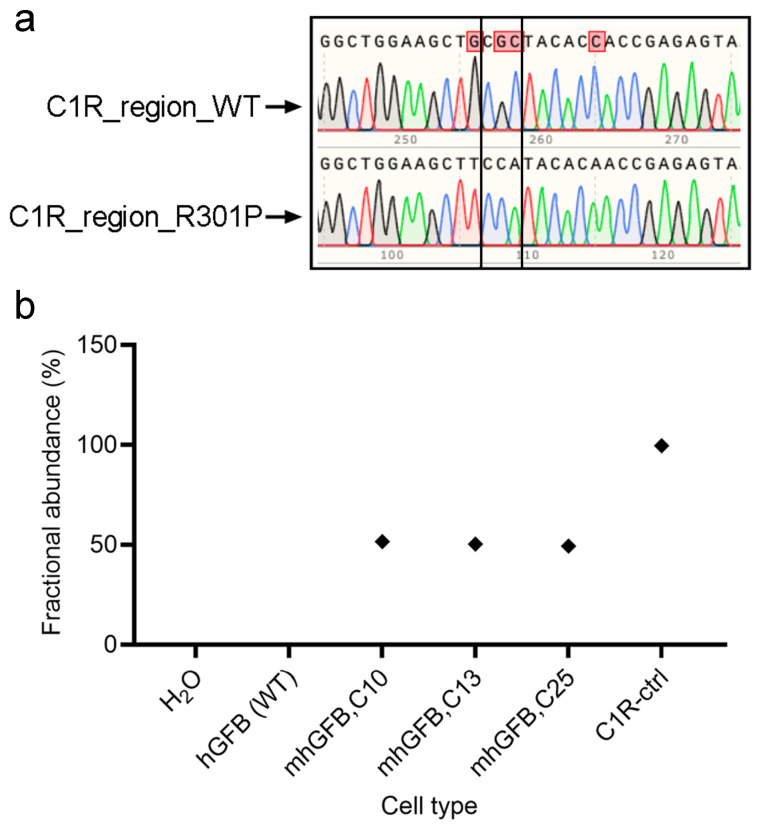
Representative Sanger sequencing chromatograms showing the edit area of C1R-R301P in one of the alleles in the mhGFB cell model (**a**). ddPCR showing the fractional abundance of the C1R^R301P/WT^-edited allele in three different mhGFB clones (**b**), representing the heterozygous mutation in mhGFBs. hGFB: hTERT-immortalized gingival fibroblasts; hGFB (WT): hGFB (wild type); mhGFBs: heterozygous C1R^R301P/WT^-mutated hGFBs; mhGFB,C10: mhGFB clone 10; mhGFB,C13: mhGFB clone 13; mhGFB,C25: mhGFB clone 25; C1R-ctrl: C1R—control.

Following treatment and expansion of the edited cell pool, single cells were isolated using fluorescence-activated cell sorting (Sony, Europe B.V., Weybridge, UK) and then expanded. Finally, the mutation was confirmed using PCR and Sanger sequencing. All three analyzed clones showed a heterozygous C1R^R301P/WT^ mutation, with one mutated allele and one wild type (Figure 1a). This finding was further validated using ddPCR, which indicated approximately 50% mutant burden in each clone (Figure 1b). Collectively, these results confirm the successful introduction of a heterozygosity of the C1R^R301P/WT^ mutation into hGFBs, creating the mhGFB cell model.

### 3.2. The mhGFBs Display Normal Cell Morphology, Growth, and Proliferation

**Figure 2:**
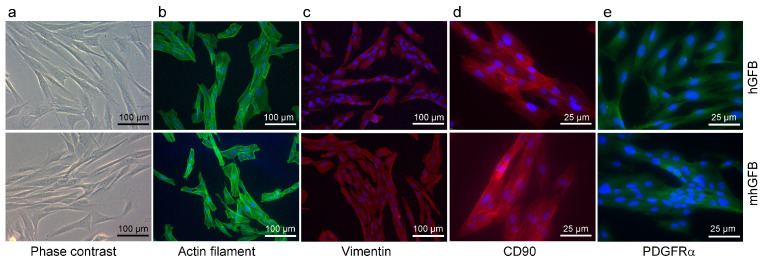
Representative photomicrographs of hGFBs and mhGFBs in 2D culture (**a**), filamentous actin (**b**), vimentin (**c**), CD90 (**d**), and PDGFRα (**e**) staining. Cell nuclei are stained in blue color with 4′,6-diamidino-2-phenylindole (DAPI).

We investigated whether the C1R^R301P/WT^ heterozygous mutation in mhGFBs affects cellular morphology and growth. Both mhGFBs and hGFBs displayed a typical fibroblast-like appearance (Figure 2). Furthermore, both cell types expressed filamentous actin, a crucial cytoskeletal component (Figure 2), and vimentin and CD90, markers typically associated with human gingival fibroblasts [36] (Figure 2). Additionally, both cell types expressed PDGFRα, another fibroblastic marker [37,38] (Figure 2). Cell nuclei of the hGFBs and mhGFBs were stained with DAPI (Figure 2). However, no significant differences in cell morphology or marker expression were observed between mhGFBs and hGFBs (Figure 2).

**Figure 3:**
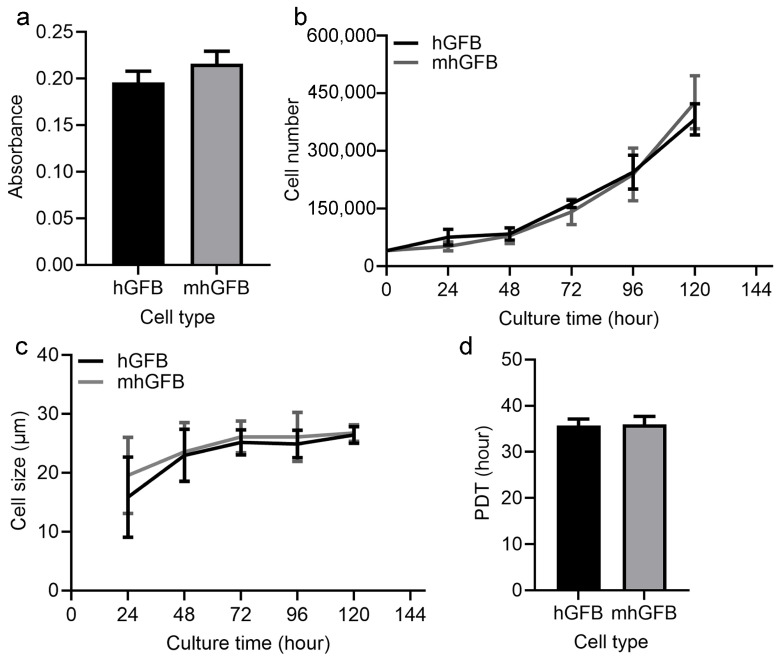
Cell proliferation (**a**), cell growth (**b**), cell size (**c**), and PDT (**d**) of the hGFBs and mhGFBs were investigated after cultured under identical conditions in 2D culture. Results are presented as mean ± SD.

Cell proliferation was examined after culturing hGFBs or mhGFBs (clone 10, clone 13, and clone 25) with FB medium in 2D cultures for 48 h. Cell proliferation assays using the MTT assay revealed no significant difference between hGFBs and mhGFBs (Figure 3a). Additionally, there was no significant variation among the three different mhGFB clones. We subsequently selected one mhGFB clone for further experiments.

Moreover, mhGFBs and hGFBs displayed comparable patterns of cell growth (Figure 3b) and cell size (Figure 3c) when cultured for up to 120 h under identical conditions. Additionally, PDT analysis after 72-h culture showed no significant difference between the two cell types (Figure 3d).

### 3.3. Differences in Messager RNA (mRNA) Expression of C1R, C1S, Procollagen α1(I), Procollagen α1(III), Procollagen α1(V), MMP-1, and TIMP-1 in mhGFBs, as Well as C1r and C1s Protein Expression

**Figure 4:**
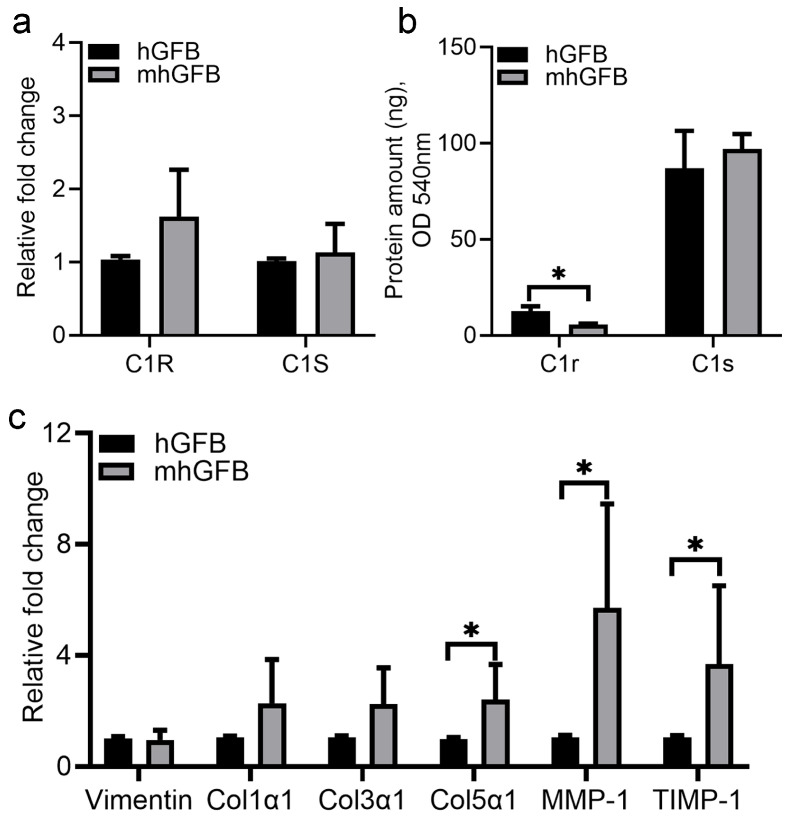
The *C1R*/*C1S* gene mRNA (**a**) and C1r/C1s protein (**b**) expressions, as well as mRNA expression of vimentin, procollagen α1(I) (Col1α1), procollagen α1(III) (Col3α1), procollagen α1(V) (Col5α1), matrix metalloproteinase-1 (MMP-1), and tissue inhibitor of metalloproteinase-1 (TIMP-1) (**c**) in hGFBs and mhGFBs after they were cultured in 2D culture for 24 h. C1R: complement component 1r; C1S: complement component 1s. Results are presented as mean ± SD. * indicates statistical significance, with *p* < 0.05.

No significant differences in *C1R* and *C1S* mRNA expression were seen in mhGFBs compared to hGFBs (Figure 4a). However, mhGFBs displayed significantly lower C1r protein levels in the culture medium (Figure 4b). Interestingly, mhGFBs exhibited a significantly higher mRNA expression of procollagen α1(V), MMP-1, and TIMP-1 compared to hGFBs (Figure 4c). Vimentin, a fibroblastic marker, and procollagen α1(I) and procollagen α1(III) did not differ significantly in mRNA expression between the two cell types (Figure 4c).

### 3.4. No Difference in Type I Collagen Staining, but a Trend Toward an Increase in Unfolded Collagens Was Observed in mhGFBs

**Figure 5:**
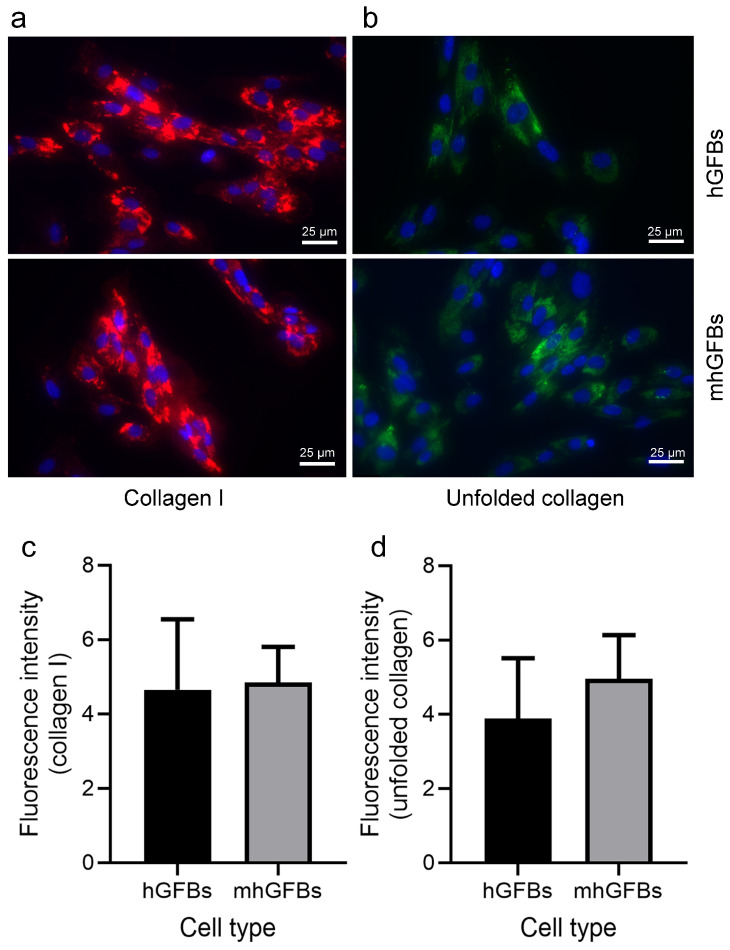
Representative images of type I collagen (**a**) and unfolded collagens (**b**) with immunocytochemical staining of hGFBs and mhGFBs after they were cultured in 2D cultures for 24 h. The fluorescence intensity of type I collagen (**c**) and unfolded collagens (**d**) was analyzed. Cell nuclei are stained with DAPI. Results are presented as mean ± SD.

Immunocytochemical staining showed no significant difference in the amount and intensity of type I collagen between mhGFBs and hGFBs (Figure 5a,c). Interestingly, the unfolded collagens, visualized using F-CHP staining, exhibited a trend toward an increase in terms of fluorescence intensity in mhGFBs (Figure 5b). However, it did not reach statistical significance when compared to hGFBs (Figure 5d).

### 3.5. Collagen Deposition Is Lower in mhGFBs

**Figure 6:**
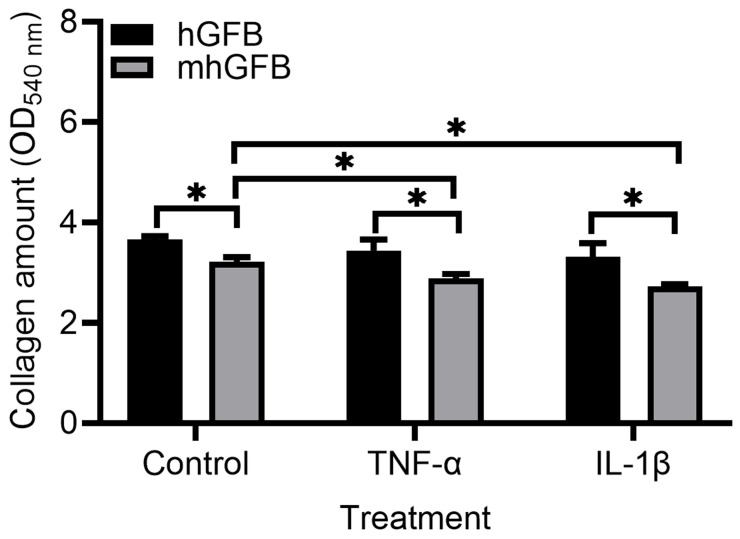
Collagen deposition in mhGFBs and hGFBs after they were cultured with FB medium (control) or treated with 50 ng/mL of TNF-α or 100 pg/mL IL-1β for 24 h in 2D culture. Data are presented as mean ± SD. * indicates statistical significance, with *p* < 0.05.

Compared to hGFBs cultured under identical conditions, mhGFBs exhibited significantly lower collagen deposition (Figure 6). This difference was maintained when the cells were treated with inflammatory cytokines TNF-α or IL-1β (Figure 6). Notably, TNF-α or IL-1β treatment further reduced collagen deposition in mhGFBs compared to untreated mhGFBs but not in hGFBs (Figure 6).

### 3.6. The Effects of Pro-Inflammatory Cytokines on the mRNA Expression of C1R, C1S, Procollagen α1(I), Procollagen α1(III), Procollagen α1(V), MMP-1, and TIMP-1 in mhGFBs

Because pro-inflammatory cytokines reduced collagen deposition in mhGFBs, we investigated if these cytokines affected gene expression of *C1R, C1S*, and collagen-related markers in mhGFBs in comparison with hGFBs.

**Figure 7:**
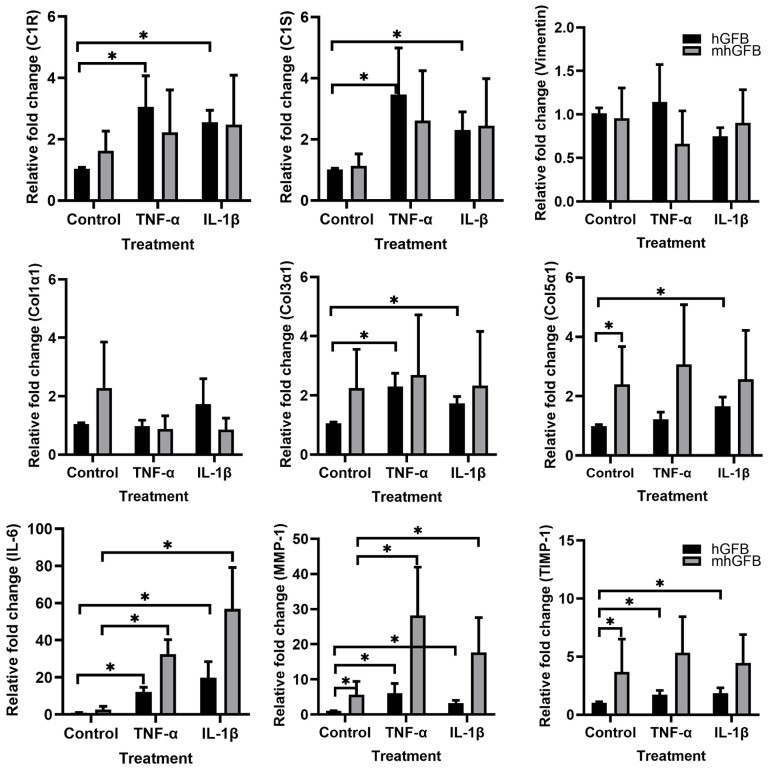
The mRNA expressions of *C1R*, *C1S*, vimentin, procollagen α1(I) (Col1α1), procollagen α1(III) (Col3α1), procollagen α1(V) (Col5α1), IL-6, MMP-1, and TIMP-1 in hGFBs and mhGFBs after they were cultured with or without 50 ng/mL of TNF-α or 100 pg/mL IL-1β in 2D culture for 24 h. Cultures without cytokine treatment served as controls. Data are presented as mean ± SD. * indicates statistical significance, with *p* < 0.05.

The hGFBs stimulated with TNF-α and/or IL-1β significantly increased *C1R, C1S*, procollagen α1(III), procollagen α1(V), MMP-1, TIMP-1, and IL-6 mRNA expression compared to controls (Figure 7). In contrast, mhGFBs only showed significant upregulation of MMP-1 and IL-6 mRNA upon stimulation with the same cytokines (Figure 7). Notably, the increase in these genes was much higher in cytokine-stimulated mhGFBs compared to cytokine-stimulated hGFBs (Figure 7).

## 4. Discussion

We successfully created a heterozygous C1R^R301P/WT^ mutation, mimicking a pEDS mutation [7], by introducing the specific C1R-R301P mutation into the hGFBs. These mutated cells, termed mhGFBs, exhibit a defined genetic makeup for C1R^R301P/WT^, characterized by one allele of the *C1R* gene carrying the mutation (C1R-R301P), while the other remains unchanged (wild type, WT). Although the prime editing efficiency was confirmed by Sanger sequencing and ddPCR to exhibit high precision of the desired mutation, the potential for off-target effects remains a consideration. Future studies incorporate whole-genome sequencing or targeted deep sequencing to confirm the specificity of genome editing.

The present results collectively indicate that the heterozygous C1R^R301P/WT^ mutation likely targets specific fibroblast functions rather than causing a global shift in their mesenchymal identity. Moreover, the observed discrepancy between *C1R*/*C1S* mRNA and C1r/C1s protein expression levels existing in the present mhGFBs demonstrates that the variant cannot integrate into the C1 complex due to impaired C1q binding, as previously reported [13].

These observations suggest that potential post-transcriptional regulatory mechanisms, such as inefficient *C1R* mRNA translation or accelerated C1r protein degradation, may underlie the discordance between unchanged *C1R* mRNA levels and significantly reduced C1r protein in mhGFBs. Post-transcriptional regulation encompasses processes, including mRNA splicing, nuclear export, stability, and translation efficiency. Inefficient translation of *C1R* mRNA potentially mediated by RNA-binding proteins or microRNAs targeting its untranslated regions could diminish C1r synthesis despite stable transcript levels, leading to lower levels in the culture medium [39,40]. Additionally, the presence of misfolded proteins may trigger proteasomal degradation pathways, leading to reduced extracellular C1r levels. The divergence between mRNA and protein levels highlights the complex regulatory mechanisms governing complement activation in periodontal health and disease.

The imbalance of C1r and C1s levels could exacerbate tissue damage by disrupting immune homeostasis. As critical components of periodontal host defense [41], their overactivation can excessively amplify inflammation, potentially leading to immunopathology [12]. As shown by Gong et al. [42] and Vila [43], specific point mutations can influence protein stability, function, or interactions with other molecules. Disease-associated mutations, particularly those affecting protein–protein interactions, can disrupt these cellular functions and drive pathogenesis [44]. In periodontal tissues, dysregulation of complement activation on host cells may provoke immune imbalance, emphasizing the need for tight regulation of synthesis and degradation pathways to prevent pathological outcomes.

Ehlers–Danlos syndrome encompasses 13 distinct subgroups, many caused by dominant-negative mutations in genes coding type I, III, and V collagens [45,46]. However, studying the impact of these mutations on collagen production in specific EDS subtypes, like pEDS, proves challenging due to the difficulty of acquiring patient gingival tissue. To address this, we developed this heterozygous C1R^R301P/WT^ mutation in hGFBs and investigated collagen biosynthesis within it.

Collagen deposition is fundamental to the cellular microenvironment, providing structure and support. Reduced collagen deposition in mhGFBs likely stems from impaired collagen synthesis due to the heterozygous C1R^R301P/WT^ mutation. This mutation likely disrupts C1r protein function, interfering with intracellular signaling pathways that regulate collagen production. Defects in collagen secretion could arise from disruptions in the transport of procollagen from the endoplasmic reticulum (ER) to the Golgi apparatus and then to the extracellular matrix (ECM) [47]. The C1R^R301P/WT^ mutation may impair vesicular transport proteins, which are essential for procollagen export from the ER. Post-translational modifications are crucial for collagen stability and function. Defective post-translational modifications can lead to misfolding and instability of collagen molecules, resulting in their deposition in the ECM [48]. The C1R^R301P/WT^ mutation can impair the modifications by disrupting the enzymes responses for collagen modifications. The ER stress occurs when there is an accumulation of misfolded or unfolded protein in the ER, triggering the unfolded protein response to restore normal function [49]. The C1R^R301P/WT^ mutation may exacerbate ER stress by producing misfolded collagen, which in turn activates the unfolded protein response that inhibits collagen synthesis. Additionally, ER stress can induce inflammation, further impacting collagen production [50]. 

Moreover, the mutation may sensitize mhGFBs to inflammation, further impacting collagen synthesis. These findings suggest that the heterozygous C1R^R301P/WT^ mutation disrupts protein stability, folding, and deposition, likely affecting protein processing rather than gene transcription, as *C1R* and *C1S* mRNA levels are normal. Upregulated procollagen α1(V) mRNA in mhGFBs, along with slightly increased procollagen α1(I) and α1(III) mRNA levels, may represent a compensatory mechanism for dysfunctional collagen.

One theory suggests that individuals with pEDS exhibit a heightened inflammatory response to the biofilm covering the tooth, contributing to rapid periodontitis progression. To investigate this, we examined the response of mhGFBs to inflammatory stimuli, mimicking the potential role of inflammation in pEDS pathogenesis. Interestingly, unlike hGFBs, mhGFBs exhibited no significant change in procollagen α1(III) and α1(V) mRNA expression upon cytokine stimulation. This suggests that the heterozygous C1R^R301P/WT^ mutation primarily disrupts collagen production in mhGFBs, likely due to protein instability and improper folding. This impairment seems independent of the inflammatory state, although mhGFBs may attempt to compensate by increasing procollagen mRNA expression.

The observed increase in MMP-1 mRNA expression and lack of TIMP-1 mRNA upregulation in cytokine-stimulated mhGFBs suggests a dysregulated inflammatory response, potentially due to altered signaling pathways, a pre-existing inflammatory state, ER stress, or post-transcriptional mechanisms. This imbalance in MMP-1/TIMP-1 expression, with increased MMP-1 production [51,52] may contribute to excessive ECM degradation in pEDS. The TIMP-1 regulates MMP-1 activity, and their balance is critical for maintaining tissue homeostasis. An imbalance favoring MMP-1 activity is associated with pathological ECM degradation [53,54].

While elevated TIMP-1 mRNA expression has been reported in periodontitis [51,55], it is believed that the host preferentially expresses TIMP-1 to counteract MMP-1 activity and prevent tissue destruction [52,56]. Interestingly, TIMP-1 mRNA expression in mhGFBs lacked a clear response to inflammatory stimuli. This suggests a potential imbalance between MMP-1 production and its inhibition in mhGFBs, potentially leading to excessive ECM degradation.

Interleukin-6 is a potent stimulator of MMP production [57,58,59]. Studies have shown that IL-6 released from fibroblasts can mediate MMP-1 upregulation [59], and IL-6 can mediate TNF-α-induced MMP-1 expression [57]. Our findings align with these established links. Furthermore, our findings suggest that the C1R^R301P/WT^ mutation may lead to an enhanced inflammatory response in mhGFBs, characterized by elevated IL-6 production. This increased IL-6 production may further stimulate MMP-1 expression and contribute to the observed imbalance, ultimately contributing to the excessive MMP-mediated tissue breakdown characteristic of pEDS.

The imbalance in MMP-1/TIMP-1 expression in mhGFBs suggests dysregulated collagen degradation. While this imbalance likely contributes to ECM degradation, it remains unclear whether C1r/C1s directly interact with ECM components to influence this process. Given the role of C1R mutations in complement activation, complement inhibitors may serve as potential therapeutic targets. Previous studies have explored complement-targeting therapies in inflammatory disorders [60,61], suggesting that inhibiting C1 activation could mitigate excessive inflammation and ECM degradation in pEDS. Future research should investigate whether complement inhibitors can restore ECM homeostasis in fibroblasts with C1R mutations.

Unlike arthrochalasia EDS, classical-vascular EDS, vascular EDS, and classic EDS, which are characterized by mutations in collagen-coding genes (col1α1, col3α1, and col5α1), pEDS is associated with mutations in the complement pathway rather than direct structural collagen defects. However, our findings suggest that the C1R^R301P/WT^ mutation indirectly influences collagen synthesis and deposition. In contrast to the severe structural deficiencies seen in classical EDS, pEDS fibroblasts exhibit altered post-translational regulation of collagen, potentially due to disrupted intracellular signaling or stress responses. Further studies are needed to determine whether this phenotype overlaps with other non-collagenous EDS variants.

This study investigates the impact of the heterozygous C1R^R301P/WT^ mutation in hGFBs, a model system that mimics the condition of patients with pEDS. While these findings offer valuable insights, this study has several limitations. (1). We initially focused on the transcriptional regulation of collagen and complement components to understand how the mutation affects *C1R/C1S* mRNA and collagen gene expression. While protein levels are the ultimate functional readout, the observed changes in gene expression provide a crucial first step in understanding the molecular mechanisms underlying the observed phenotype. Including protein analysis using Western blot or ELISA assays would indeed deepen our study. (2). A limitation of this study is the use of immortalized gingival fibroblasts, which, while providing a stable and reproducible model, may not fully recapitulate the behavior of primary fibroblasts in vivo. The present findings from hGFBs should be interpreted with caution, as immortalization may influence certain cellular behaviors, potentially impacting translational relevance. To address this limitation, future studies should validate these results in primary gingival fibroblasts derived from pEDS patients or appropriate animal models to better understand the pathophysiology of C1R mutation in periodontal tissues. (3). The sample size is relatively small, and the investigation focuses on a single C1R mutation. To further elucidate the role of C1R mutation in the pathogenesis of pEDS, future research should investigate protein levels, activity, and collagen degradation in a larger cohort of hGFBs, including those with diverse C1R mutations. Additionally, the functional consequences of other C1R mutations on C1r protein function and collagen metabolism warrant further investigation.

## 5. Conclusions

To our knowledge, this study is the first to investigate the impact of a specific heterozygous C1R^R301P/WT^ mutation on hGFBs, which mimics key aspects of pEDS. Our findings demonstrate that this mutation disrupts collagen homeostasis and inflammatory signaling in hGFBs. Specifically, the mutation led to increased MMP-1 and IL-6 expression, reduced C1r protein levels, and impaired collagen deposition, highlighting its potential role in pEDS pathology. The unchanged type I collagen, a trend of increased unfolded collagen and reduced collagen deposition, indicates that the C1R^R301P/WT^ mutation affects collagen processing and deposition rather than the total collagen production, as evidenced by differential collagen gene expression. This multifaceted impact underscores the complexity of collagen biology and the need for comprehensive analyses at both the protein and gene expression levels in the future experiment.

Future research should explore whether complement inhibitors can mitigate the effects of C1R mutations on collagen metabolism and inflammatory pathways. Additionally, validating these findings in primary patient-derived fibroblasts or animal models will provide further insights into the pathophysiology of pEDS and potential therapeutic strategies.

## Figures and Tables

**Table 1:** The list of human primers used in RT-qPCR.

Gene	Primer Pairs (5′→ 3′)	Product Size (bp)
RPLP0	F: AGATGCAGCAGATCCGCAT R: GTGGTGATACCTAAAGCCTG	319
*C1R*	F: TTCCCCAAGCCTTACCCCAA R: GCTGGAAGACGAGCTTCACC	85
*C1S*	F: ACTGTGCGTATGACTCAGTGC R: GGGGATTGTTACTGCTCCTCT	84
Vimentin	F: CCTGCAATCTTTCAGACAGG R: CTCCTGGATTTCCTCTTCGT	127
Col1α1	F: GAACGCGTGTCATCCCTTGT R: GAACGAGGTAGTCTTTCAGCAACA	94
Col3α1	F: TGGTCTGCAAGGAATGCCTGGA R: TCTTTCCCTGGGACACCATCAG	108
Col5α1	F: CTCCCTGCTTTCTTTATCCT R: GAGTGTGCTTGGC TATCCTG	108
IL-6	F: TGGCTGAAAAAGATGGATGCT R: TCTGCACAGCTCTGGCTTGT	150
MMP-1	F: TCGATGCTGCTCTTTCTGAG R: AACTTTGTGGCCAATTCCAG	149
TIMP-1	F: AGTGATGTGCAAGAGTCCATCCTG R: CAGCGTTA GAGATCAAGATGACCA	184

RPLP0: ribosomal protein lateral stalk subunit P0; *C1R*: complement component 1r; *C1S*: complement component 1s; Col1α1: procollagen α1(I); Col3α1: procollagen α1(III); Col5α1: procollagen α1(V); IL-6: interleukin-6; MMP-1: matrix metalloproteinase-1; TIMP-1: tissue inhibitor of metalloproteinase-1.

## Data Availability

All data used in this study are available on request from the corresponding author.

## Abbreviations

The following abbreviations are used in this manuscript:

hGFBs	human TERT-immortalized gingival fibroblasts
mhGFBs	heterozygous C1R^R301P/WT^-mutated hGFBs
MMP-1	matrix metalloproteinase-1
IL-6	interleukin-6
TIMP-1	tissue inhibitor of metalloproteinase-1
C1r (C1R)	complement component 1r (1R)
C1s (C1S)	complement component 1r (1S)
CD90	cluster of differentiation 90
Col1α1	procollagen α1(I)
Col3 α1	procollagen α1(III)
Col5 α1	procollagen α1(V)
pEDS	periodontal Ehlers–Danlos syndrome
FB	fibroblast
ICC	immunocytochemical staining
PDT	population doubling time
PDGFRα	platelet-derived growth factor receptor alpha
TNF-α	tumor necrosis factor alpha
IL-1β	interleukin-1 beta
pegRNA	prime editing guide RNA
PCR	polymerase chain reaction
ddPCR	droplet digital PCR
MTT	3-(4,5-dimethylthiazol-2-yl)-2,5-diphenyltetrazolium bromide
F-CHP	fluorescence-collagen hybridizing peptide
DAPI	4′6′-Diamidino-2-phenylindole dihydrochloride
ELISA	enzyme-like immunosorbent assay
RT-qPCR	real-time quantitative reverse transcription polymerase chain reaction
RPLP0	ribosomal protein lateral stalk subunit P0
RNA	ribonucleic acid
mRNA	message RNA
ECM	extracellular matrix
ER	endoplasmic reticulum

## References

[1] Islam M., Chang C., Gershwin M.E. (2021). Ehlers-Danlos Syndrome: Immunologic contrasts and connective tissue comparisons. J. Transl. Autoimmun..

[2] Kapferer-Seebacher I., Lundberg P., Malfait F., Zschocke J. (2017). Periodontal manifestations of Ehlers-Danlos syndromes: A systematic review. J. Clin. Periodontol..

[3] Albandar J.M., Susin C., Hughes F.J. (2018). Manifestations of systemic diseases and conditions that affect the periodontal attachment apparatus: Case definitions and diagnostic considerations. J. Periodontol..

[4] Kapferer-Seebacher I., Oakley-Hannibal E., Lepperdinger U., Johnson D., Ghali N., Brady A.F., Sobey G., Zschocke J., van Dijk F.S. (2021). Prospective clinical investigations of children with periodontal Ehlers-Danlos syndrome identify generalized lack of attached gingiva as a pathognomonic feature. Genet. Med..

[5] Kapferer-Seebacher I., van Dijk F.S., Zschocke J., Adam M.P., Feldman J., Mirzaa G.M., Pagon R.A., Wallace S.E., Bean L.J.H., Gripp K.W., Amemiya A. (2021). Periodontal Ehlers-Danlos Syndrome Synonyms: EDS Type VIII, pEDS. GeneReviews [Internet].

[6] Martins R.S., Muniz F.W.M.G., Gondim J.O., Maurique L.S., Nolasco-Lopes C.M., Oliveira B.M., Carvalho R.S. (2023). Periodontal Ehlers-Danlos syndrome in early childhood: A case report of loss of deciduous teeth. J. Indian Soc. Periodontol..

[7] Kapferer-Seebacher I., Pepin M., Werner R., Aitman T.J., Nordgren A., Stoiber H., Thielens N., Gaboriaud C., Amberger A., Schossig A. (2016). Periodontal Ehlers-Danlos Syndrome Is Caused by Mutations in C1R and C1S, which Encode Subcomponents C1r and C1s of Complement. Am. J. Hum. Genet..

[8] Reinstein E., DeLozier C.D., Simon Z., Bannykh S., Rimoin D.L., Curry C.J. (2013). Ehlers-Danlos syndrome type VIII is clinically heterogeneous disorder associated primarily with periodontal disease, and variable connective tissue features. Eur. J. Hum. Genet..

[9] Rinner A., Zschocke J., Schossig A., Grobner R., Strobl H., Kapferer-Seebacher I. (2018). High risk of peri-implant disease in periodontal Ehlers-Danlos Syndrome. A case series. Clin. Oral Implant. Res..

[10] Nguyen V.C., Tosi M., Gross M.S., Cohen-Haguenauer O., Jegou-Foubert C., de Tand M.F., Meo T., Frezal J. (1988). Assignment of the complement serine protease genes C1r and C1s to chromosome 12 region 12p13. Hum. Genet..

[11] Rahman N., Dunstan M., Teare M.D., Hanks S., Douglas J., Coleman K., Bottomly W.E., Campbell M.E., Berglund B., Nordenskjöld M. (2003). Ehlers-Danlos syndrome with severe early-onset periodontal disease (EDS-VIII) is a distinct, heterogeneous disorder with one predisposition gene at chromosome 12p13. Am. J. Hum. Genet..

[12] Almitairi J.O.M., Venkatraman Girija U., Furze C.M., Simpson-Gray X., Badakshi F., Marshall J.E., Schwaeble W.J., Mitchell D.A., Moody P.C.E., Wallis R. (2018). Structure of the C1r-C1s interaction of the C1 complex of complement activation. Proc. Natl. Acad. Sci. USA.

[13] Gröbner R., Kapferer-Seebacher I., Amberger A., Redolfi R., Dalonneau F., Björck E., Milnes D., Bally I., Rossi V., Thielens N. (2019). C1R Mutations Trigger Constitutive Complement 1 Activation in Periodontal Ehlers-Danlos Syndrome. Front. Immunol..

[14] Noris M., Remuzzi G. (2013). Overview of complement activation and regulation. Semin. Nephrol..

[15] Bally I., Dalonneau F., Chouquet A., Gröbner R., Amberger A., Kapferer-Seebacher I., Stoiber H., Zschocke J., Thielens N.M., Rossi V. (2019). Two Different Missense C1S Mutations, Associated to Periodontal Ehlers-Danlos Syndrome, Lead to Identical Molecular Outcomes. Front. Immunol..

[16] Amberger A., Pertoll J., Traunfellner P., Kapferer-Seebacher I., Stoiber H., Klimaschewski L., Thielens N., Gaboriaud C., Zschocke J. (2023). Degradation of collagen I by activated C1s in periodontal Ehlers-Danlos Syndrome. Front. Immunol..

[17] Chalak M., Hesaraki M., Mirbahari S.N., Yeganeh M., Abdi S., Rajabi S., Hemmatzadeh F. (2024). Cell Immortality: In Vitro Effective Techniques to Achieve and Investigate Its Applications and Challenges. Life.

[18] Evtushenko N.A., Beilin A.K., Dashinimaev E.B., Ziganshin R.H., Kosykh A.V., Perfilov M.M., Rippa A.L., Alpeeva E.V., Vasiliev A.V., Vorotelyak E.A. (2021). hTERT-Driven Immortalization of RDEB Fibroblast and Keratinocyte Cell Lines Followed by Cre-Mediated Transgene Elimination. Int. J. Mol. Sci..

[19] Nogueira L.S., Vasconcelos C.P., Mitre G.P., Bittencourt L.O., Placa J.R., Kataoka M.S.D.S., Pinheiro J.J.V., Garlet G.P., De Oliveira E.H.C., Lima R.R. (2021). Gene Expression Profile in Immortalized Human Periodontal Ligament Fibroblasts Through hTERT Ectopic Expression: Transcriptome and Bioinformatic Analysis. Front. Mol. Biosci..

[20] Sadiq A., Khumalo N.P., Bayat A. (2024). Development and validation of novel keloid-derived immortalized fibroblast cell lines. Front. Immunol..

[21] Chen P.J., Hussmann J.A., Yan J., Knipping F., Ravisankar P., Chen P.F., Chen C., Nelson J.W., Newby G.A., Sahin M. (2021). Enhanced prime editing systems by manipulating cellular determinants of editing outcomes. Cell.

[22] Nelson J.W., Randolph P.B., Shen S.P., Everette K.A., Chen P.J., Anzalone A.V., An M., Newby G.A., Chen J.C., Hsu A. (2022). Engineered pegRNAs improve prime editing efficiency. Nat. Biotechnol..

[23] Agudelo D., Duringer A., Bozoyan L., Huard C.C., Carter S., Loehr J., Synodinou D., Drouin M., Salsman J., Dellaire G. (2017). Marker-free coselection for CRISPR-driven genome editing in human cells. Nat. Methods.

[24] Levesque S., Mayorga D., Fiset J.P., Goupil C., Duringer A., Loiselle A., Bouchard E., Agudelo D., Doyon Y. (2022). Marker-free co-selection for successive rounds of prime editing in human cells. Nat. Commun..

[25] Tullberg-Reinert H., Jundt G. (1999). In situ measurement of collagen synthesis by human bone cells with a sirius red-based colorimetric microassay: Effects of transforming growth factor beta2 and ascorbic acid 2-phosphate. Histochem. Cell Biol..

[26] Ferrà-Cañellas M.D.M., Munar-Bestard M., Garcia-Sureda L., Lejeune B., Ramis J.M., Monjo M. (2021). BMP4 micro-immunotherapy increases collagen deposition and reduces PGE2 release in human gingival fibroblasts and increases tissue viability of engineered 3D gingiva under inflammatory conditions. J. Periodontol..

[27] Koskinen Holm C., Qu C. (2022). Engineering a 3D In Vitro Model of Human Gingival Tissue Equivalent with Genipin/Cytochalasin D. Int. J. Mol. Sci..

[28] Yu L., Shen H.J., Ren X.H., Wang A.Q., Zhu S., Zheng Y.F., Wang X.L. (2021). Multi-omics analysis reveals the interaction between the complement system and the coagulation cascade in the development of endometriosis. Sci. Rep..

[29] Bhardwaj M., Sen S., Chosdol K., Bakhshi S., Pushker N., Sharma A., Kashyap S., Bajaj M., Singh V.K. (2020). Vimentin overexpression as a novel poor prognostic biomarker in eyelid sebaceous gland carcinoma. Br. J. Ophthalmol..

[30] Kim M.S., Han J.Y., Kim S.H., Kim H.Y., Jeon D., Lee K. (2018). Polyhexamethylene guanidine phosphate induces IL-6 and TNF-α expression through JNK-dependent pathway in human lung epithelial cells. J. Toxicol. Sci..

[31] Song P., Jo H.S., Shim W.S., Kwon Y.W., Bae S., Kwon Y., Azamov B., Hur J., Lee D., Ryu S.H. (2021). Emodin induces collagen type I synthesis in Hs27 human dermal fibroblasts. Exp. Ther. Med..

[32] Ren W.M., Zhang Y.Y., Zhang L.Y., Lin Q.B., Zhang J.G., Xu G.X. (2018). Overexpression of collagen type V 1 chain in human breast invasive ductal carcinoma is mediated by TGF-1. Int. J. Oncol..

[33] Tanigawa S., Aida Y., Kawato T., Honda K., Nakayama G., Motohashi M., Suzuki N., Ochiai K., Matsumura H., Maeno M. (2011). Interleukin-17F affects cartilage matrix turnover by increasing the expression of collagenases and stromelysin-1 and by decreasing the expression of their inhibitors and extracellular matrix components in chondrocytes. Cytokine.

[34] Kaitainen S., Mähönen A.J., Lappalainen R., Kröger H., Lammi M.J., Qu C.J. (2013). TiO_2_-coating promotes human mesenchymal stem cell proliferation without the loss of their capacity for chondrogenic differentiation. Biofabrication.

[35] Pfaffl M.W. (2001). A new mathematical model for relative quantification in real-time RT-PCR. Nucleic Acids Res..

[36] Palkowitz A.L., Tuna T., Bishti S., Boke F., Steinke N., Muller-Newen G., Wolfart S., Fischer H. (2021). Biofunctionalization of Dental Abutment Surfaces by Crosslinked ECM Proteins Strongly Enhances Adhesion and Proliferation of Gingival Fibroblasts. Adv. Healthc. Mater..

[37] Buechler M.B., Turley S.J. (2018). A short field guide to fibroblast function in immunity. Semin. Immunol..

[38] Schuster R., Rockel J.S., Kapoor M., Hinz B. (2021). The inflammatory speech of fibroblasts. Immunol. Rev..

[39] Laurent J.M., Vogel C., Kwon T., Craig S.A., Boutz D.R., Huse H.K., Nozue K., Walia H., Whiteley M., Ronald P.C. (2010). Protein abundances are more conserved than mRNA abundances across diverse taxa. Proteomics.

[40] Spangenberg L., Correa A., Dallagiovanna B., Naya H. (2013). Role of alternative polyadenylation during adipogenic differentiation: An in silico approach. PLoS ONE.

[41] Pouw R.B., Ricklin D. (2021). Tipping the balance: Intricate roles of the complement system in disease and therapy. Semin. Immunopathol..

[42] Gong J.T., Wang J.X., Zong X.Z., Ma Z.Q., Xu D. (2023). Prediction of protein stability changes upon single-point variant using 3D structure profile. Comput. Struct. Biotechnol. J..

[43] Vila J.A. (2022). Proteins’ Evolution upon Point Mutations. ACS Omega.

[44] Fragoza R., Das J., Wierbowski S.D., Liang J., Tran T.N., Liang S., Beltran J.F., Rivera-Erick C.A., Ye K., Wang T.Y. (2019). Extensive disruption of protein interactions by genetic variants across the allele frequency spectrum in human populations. Nat. Commun..

[45] Malfait F., Francomano C., Byers P., Belmont J., Berglund B., Black J., Bloom L., Bowen J.M., Brady A.F., Burrows N.P. (2017). The 2017 international classification of the Ehlers-Danlos syndromes. Am. J. Med. Genet. C Semin. Med. Genet..

[46] Mao J.R., Bristow J. (2001). The Ehlers-Danlos syndrome: On beyond collagens. J. Clin. Investig..

[47] Zhang Z., Luo S., Barbosa G.O., Bai M., Kornberg T.B., Ma D.K. (2021). The conserved transmembrane protein TMEM-39 coordinates with COPII to promote collagen secretion and regulate ER stress response. PLoS Genet..

[48] Tvaroska I. (2024). Glycosylation Modulates the Structure and Functions of Collagen: A Review. Molecules.

[49] Besio R., Garibaldi N., Leoni L., Cipolla L., Sabbioneda S., Biggiogera M., Mottes M., Aglan M., Otaify G.A., Temtamy S.A. (2019). Cellular stress due to impairment of collagen prolyl hydroxylation complex is rescued by the chaperone 4-phenylbutyrate. Dis. Model. Mech..

[50] Li C., Liu Y., Li Y., Tai R., Sun Z., Wu Q., Liu Y., Sun C. (2021). Collagen XV Promotes ER Stress-Induced Inflammation through Activating Integrin beta1/FAK Signaling Pathway and M1 Macrophage Polarization in Adipose Tissue. Int. J. Mol. Sci..

[51] Kubota T., Itagaki M., Hoshino C., Nagata M., Morozumi T., Kobayashi T., Takagi R., Yoshie H. (2008). Altered gene expression levels of matrix metalloproteinases and their inhibitors in periodontitis-affected gingival tissue. J. Periodontol..

[52] Kubota T., Nomura T., Takahashi T., Hara K. (1996). Expression of mRNA for matrix metalloproteinases and tissue inhibitors of metalloproteinases in periodontitis-affected human gingival tissue. Arch. Oral Biol..

[53] Soell M., Elkaim R., Tenenbaum H. (2002). Cathepsin C, matrix metalloproteinases, and their tissue inhibitors in gingiva and gingival crevicular fluid from periodontitis-affected patients. J. Dent. Res..

[54] Wang L., Almqvist K.F., Veys E.M., Verbruggen G. (2002). Control of extracellular matrix homeostasis of normal cartilage by a TGFβ autocrine pathway.: Validation of flow cytometry as a tool to study chondrocyte metabolism. Osteoarthr. Cartil..

[55] Kubota T., Matsuki Y., Nomura T., Hara K. (1997). In situ hybridization study on tissue inhibitors of metalloproteinases (TIMPs) mRNA-expressing cells in human inflamed gingival tissue. J. Periodontal Res..

[56] Nomura T., Takahashi T., Hara K. (1993). Expression of TIMP-1, TIMP-2 and collagenase mRNA in periodontitis-affected human gingival tissue. J. Periodontal Res..

[57] Du G.L., Liu C.X., Li X.O., Chen W.Y., He R., Wang X.J., Feng P.F., Lan W.W. (2016). Induction of matrix metalloproteinase-1 by tumor necrosis factor-α is mediated by interleukin-6 in cultured fibroblasts of keratoconus. Exp. Biol. Med..

[58] Li Y., Samuvel D.J., Sundararaj K.P., Lopes-Virella M.F., Huang Y. (2010). IL-6 and high glucose synergistically upregulate MMP-1 expression by U937 mononuclear phagocytes via ERK1/2 and JNK pathways and c-Jun. J. Cell Biochem..

[59] Sundararaj K.P., Samuvel D.J., Li Y., Sanders J.J., Lopes-Virella M.F., Huang Y. (2009). Interleukin-6 released from fibroblasts is essential for up-regulation of matrix metalloproteinase-1 expression by U937 macrophages in coculture: Cross-talking between fibroblasts and U937 macrophages exposed to high glucose. J. Biol. Chem..

[60] Davis A.E., Mejia P., Lu F. (2008). Biological activities of C1 inhibitor. Mol. Immunol..

[61] Ye J., Yang P., Yang Y., Xia S. (2022). Complement C1s as a diagnostic marker and therapeutic target: Progress and propective. Front. Immunol..

